# Therapy duration and long-term outcomes in extra-pulmonary tuberculosis

**DOI:** 10.1186/1471-2334-14-115

**Published:** 2014-03-01

**Authors:** Tobias Pusch, Jotam G Pasipanodya, Ronald G Hall, Tawanda Gumbo

**Affiliations:** 1 Department of Medicine, University of Texas Southwestern Medical Center, Dallas, USA; 2 Department of Pharmacy Practice, Texas Tech University Health Sciences Center, School of Pharmacy, 4500 Lancaster, Dallas, Texas 75216, USA; 3 Office of Global Health, University of Texas Southwestern Medical Center, 5323 Harry Hines Blvd, Dallas, Texas 75390-8504, USA

**Keywords:** Extra-pulmonary tuberculosis, Therapy duration, Survival, Peritoneal, Meningitis

## Abstract

**Background:**

Tuberculosis is classified as either pulmonary or extra-pulmonary (EPTB). While much focus has been paid to pulmonary tuberculosis, EPTB has received scant attention. Moreover, EPTB is viewed as one wastebasket diagnosis, as “the other” which is not pulmonary.

**Methods:**

This is a retrospective cohort study of all patients treated for EPTB in the state of Texas between January 2000 and December 2005, who had no pulmonary disease. Clinical and epidemiological factors were abstracted from electronic records of the Report of Verified Case of Tuberculosis. The long-term outcome, which is death by December 2011, was established using the Social Security Administration Death Master File database. Survival in EPTB patients was compared to those with latent tuberculosis, as well as between different types of EPTB, using Cox proportional hazard models. A hybrid of the machine learning method of classification and regression tree analyses and standard regression models was used to identify high-order interactions and clinical factors predictive of long-term all-cause mortality.

**Results:**

Four hundred and thirty eight patients met study criteria; the median study follow-up period for the cohort was 7.8 (inter-quartile range 6.0-10.1) years. The overall all-cause mortality rate was 0.025 (95% confidence interval [CI]: 0.021-0.030) per 100 person-year of follow-up. The significant predictors of poor long-term outcome were age (hazard ratio [HR] for each year of age-at-diagnosis was 1.05 [CI: 1.04-1.06], treatment duration, type of EPTB and HIV-infection (HR = 2.16; CI: 1.22, 3.83). Mortality in genitourinary tuberculosis was no different from latent tuberculosis, while meningitis had the poorest long-term outcome of 46.2%. Compared to meningitis the HR for death was 0.50 (CI: 0.27-0.91) for lymphatic disease, 0.42 (CI: 0.21-0.81) for bone/joint disease, and 0.59 (CI: 0.27-1.31) for peritonitis. The relationship between mortality and therapy duration for each type of EPTB was a unique “V” shaped curve, with the lowest mortality observed at different therapy durations for each, beyond which mortality increased.

**Conclusions:**

EPTB is comprised of several different diseases with different outcomes and durations of therapy. The “V” shaped relationship between therapy duration and outcome leads to the hypothesis that longer duration of therapy may lead to higher patient mortality.

## Background

Tuberculosis (TB) affects 8.7 million people each year; while 1 in every 3 human beings on earth has been exposed to *Mycobacterium tuberculosis* and has latent tuberculosis infection [1]. The vast majority of research on tuberculosis has focused on pulmonary disease, with efforts to shorten therapy duration. Meanwhile, the rate of “extra-pulmonary” tuberculosis (EPTB) has increased from one in every 25 TB patients in 1995 to about one in every five TB patients in 2002, and one in every three TB patients in 2011 [2,3]. More than 50% of HIV-infected patients with tuberculosis present with EPTB. Patients using tumor necrosis factor-α inhibitors for autoimmune diseases have a 4-5 fold increase in the rate of active tuberculosis compared with non-users, and have EPTB rates similar to those with AIDS [4]. The recommended treatment for drug-susceptible EPTB is with isoniazid, rifampin, ethambutol and pyrazinamide for 6 months, with the exception of tuberculous meningitis which is treated with 9 to 12 months of therapy [5]. In general, extra-pulmonary tuberculosis in adult patients has been lumped aside in a wastebasket category. Meningitis is a notable exception [6-10]. Dutt, Moers and Stead studied the outcomes over an average period of 3 years post treatment in 219 EPTB the early 1980s [11]. However, this cohort of patients was treated with a regimen of isoniazid and rifampin alone, not the standard four drug regimen used today. In a second study, Kwara et al examined data on 126 patients with EPTB at the dawn of the AIDS pandemic, and found that the short-term mortality during treatment of EPTB was associated with HIV-infection, meningeal tuberculosis and disseminated disease [12]. Long-term outcomes were not reported. Thus, the long-term outcome in EPTB patients “adequately treated” with the current standard regimen is unknown, so that it is unclear if standard short course therapy is effective in reducing long-term mortality. We have developed methods to use the Texas electronic database to examine long-term outcomes such as death in tuberculosis patients [6]. This study is focused on the evaluation of long-term outcomes in patients with EPTB, and the demographic and therapy factors predictive of poor long-term outcome.

## Methods

### Study design

We performed a retrospective cohort study in which all patients treated for EPTB between January 1^st^, 2000, and December 31^st^, 2005, had records examined for clinical and epidemiological factors. The main long-term outcome, which is all-cause mortality by December 31, 2011, was then established for each patient. We compared all-cause mortality rates between EPTB and of age-adjusted Texas population as well as of another comparable cohort of patients with latent tuberculosis treated during the same period [13,14]. The latent tuberculosis patient cohort was previously reported by us [13,14], while the Texas age-adjusted all-cause population mortality rates are published annually by the Centers for Disease Control and Prevention (CDC) [15].

### Study population and setting

All adult patients who were treated for EPTB and recorded in the electronic State of Texas surveillance database, as defined by CDC’s Report of Verified Case of Tuberculosis manual were eligible for the study. We excluded persons ≤18 years-old, since pediatric tuberculosis disease is different from that in adults [16]. Texas has 254 counties, which report to the Texas Department of State Health Services. The Texas Department of State Health Services requires health care workers to report an active tuberculosis case within one working day and latent tuberculosis within one week to the local county health authority. All tuberculosis patients in Texas are treated using CDC recommended regimens and the directly observed therapy strategy (DOTS) [5]. All latent tuberculosis patients are offered free isoniazid treatments for at least six months [17].

### IRB and regulatory permissions

The original study was to identify the effect of factors such as weight and other covariates on long term mortality outcome of tuberculosis patients in Texas. The study was reviewed and approved by the Institutional Review Boards (IRB) of UT Southwestern Medical Center (IRB# 122008-020) and the Texas State Department of Human and Health Services: [(IRB #09-021) Principal Investigator: Ronald Hall].

### Definitions and classifications utilized

Tuberculosis case definitions and classification as EPTB were based on generic definitions included in the Report of Verified Case of Tuberculosis manual. An episode of microbiologically proven tuberculosis disease is defined as: (1) isolation of *M. tuberculosis* by culture from a clinical specimen, or (2) demonstration of *M. tuberculosis* in a clinical specimen by nucleic acid amplification test, or (3) demonstration of acid-fast bacilli in a clinical specimen when a culture has not been or cannot be obtained or is falsely negative or contaminated. Patients were considered to have EPTB if radiographic imaging or clinical samples taken from disease sites were consistent with tuberculosis, and/or if clinical assessment was highly suggestive of tuberculosis. EPTB comprised any extra-pulmonary disease site: lymphatic, bone and/or joint, genitourinary, meningeal, peritoneal, and unclassified EPTB sites listed as “other” in the Report of Verified Case of Tuberculosis. For the purpose of this study, we excluded patients who had both EPTB and pulmonary tuberculosis, including any intra-thoracic disease or pleural tuberculosis or miliary tuberculosis. Previous anti-tuberculosis therapy refers to patients who reported receipt of treatment for tuberculosis in the medical history.

All-cause mortality was chosen as the primary endpoint of interest since the exact causes of death were not ascertained for each patient. The dates of when patients started and stopped therapy are captured in the Report of Verified Case of Tuberculosis, and the duration of therapy computed using these dates for all patients in whom therapy was not interrupted, and rounded to the nearest month. Based on proposals in the published literature, we examined the following categorized therapy durations defined *a priori*: <6-months, 6-months, 6-10 months and > -10 months [5,11,12]. In addition, we took an unbiased approach, specified no categories, and identified cut-off levels based on the data by employing classification and regression analysis (CART). Additionally, based on patients’ case completion reports on follow-up, comparisons were made between patient groups using “reasons therapy stopped”.

### Database and data collection methods

We performed a retrospective review of all electronic medical records of tuberculosis patients recorded in the Report of Verified Cases of Tuberculosis report for the State of Texas for the years 2000 to 2005. The database includes epidemiological, demographic, clinical and laboratory data. Each case is reviewed and validated at each administrative level. Each patient’s death was ascertained by querying the patient’s record against an updated electronic copy of the Social Security Administration (SSA) Death Master File database. The US Department of Commerce’s National Technical Information Service verifies records of deaths in the US and administers the SSA Death Master File database to prevent identity fraud. The SSA Death Master File database is widely used for various diseases and population-level studies; its methods are well validated and it has an accuracy of 93-96% [18-20]. Mortality ascertainment was by “exact matches”, i.e., sharing locality, demographic factors and period of care, with subject record between the SSA Death Master File database and our Texas tuberculosis file, using previously described methods [6]. Since the SSA Death Master File database comprises of deceased persons formerly eligible for legal work authorization, we restricted our analysis to US citizens and legal residents.

### Statistical analysis

We employed machine learning models that have high predictive accuracy in identifying both high-order complexity non-linear and linear interactions to identify risk factors of poor long-term outcome in EPTB [21-27]. We did not assume linear interactions between risk factors and clinical outcomes since most biological processes are inherently non-linear and their interactions are obviously non-linear. We decided *a priori* to employ *hybrid models* that included data mining and standard statistical methods. Classification and regression tree (CART) analyses were performed using the Salford Predictive Modeling software. The CART modelling approach was used to rank important predictors of death and to identify complex interaction between predictors, and to identify optimal thresholds for classifying patients by mortality if a predictor was a continuous variable. CART, a binary recursive portioning technique, splits predictors at each node into homogenous groups based on assigned penalty rules. By employing the GINI coefficient, the automated software can identify optimal cut-off points that maximize penalty rules for continuous variables. Most important variables for the optimal tree are scored and selected then ranked based on the first node (which is also called the root node). The variable used in the root node is scored 100%. Ten-fold cross-validation methods were used to determine the accuracy of model approach. In this process, the learning database is randomly split into ten sets, after which CART is run to estimate what the error rate of a sub-tree would be if you had a test database, thereby obviating the need for a validation database.

Cox proportional analyses were used to compute mortality hazard rates and to identify independent predictors of survival among the different patient groups, including those who had successfully completed therapy. Next, a comparison of patients’ demographic, clinical and treatment outcomes between major anatomic sites of tuberculosis disease (hereby termed EPTB syndromes) was made using Pearson’s chi-square test and when appropriate Fischer’s exact tests for categorical data. Age was modeled both as discrete (for ease of comparison with general population survival which is reported in 10-year increments) and as a continuous variable (for comparison between patient groups). Otherwise, continuous data was analyzed by means of Kruskal-Wallis tests for non-normally distributed data and analysis of variance or Students’ *T*-tests for otherwise. Kaplan-Meier plots together with the log-rank test were used to examine and to compare cumulative survival between groups. All analyses were two-sided with p ≤ 0.05 significance levels and were performed using STATA (College Station, TX) version 12.

## Results

**Figure 1:**
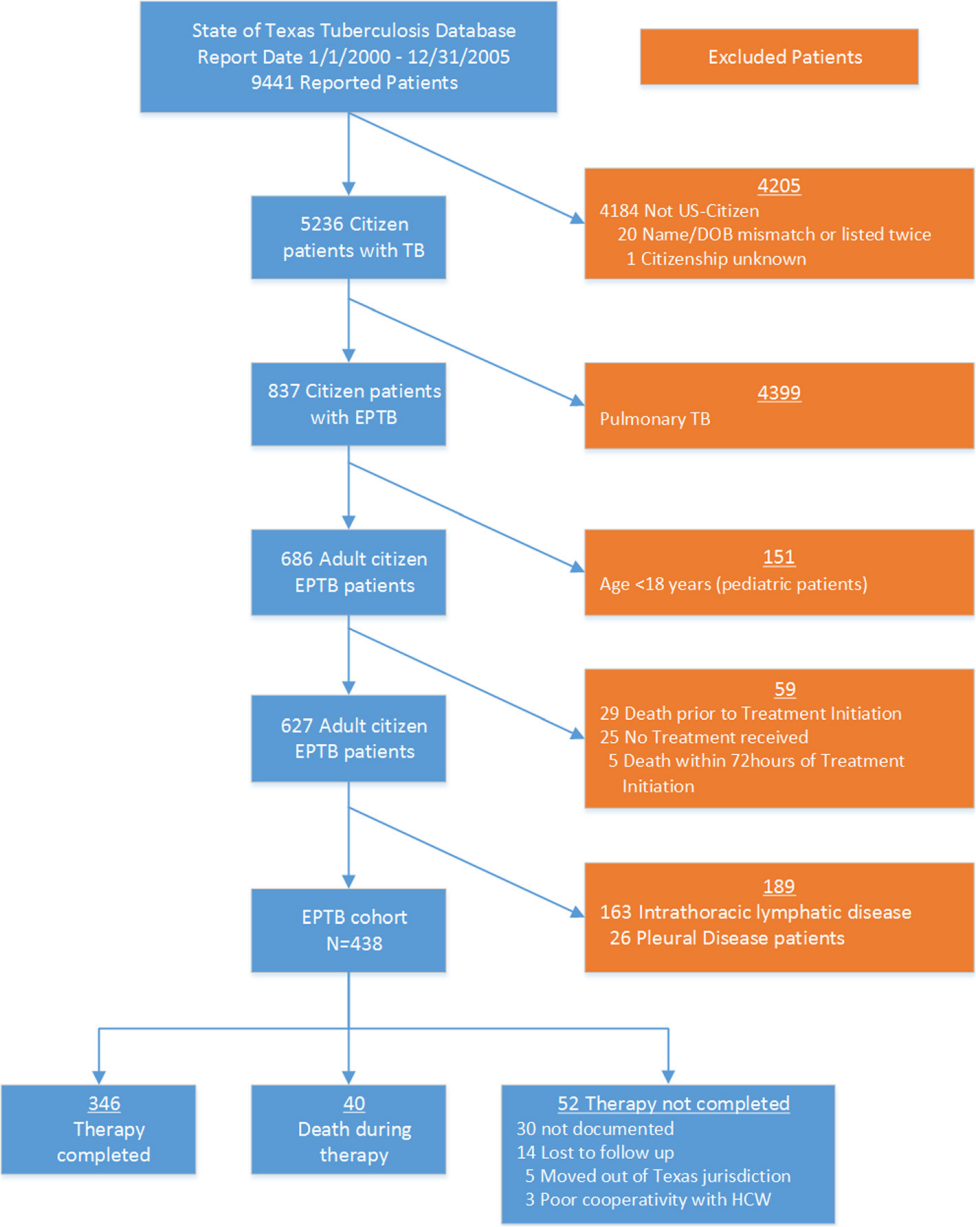
Study enrollment outline.

**Figure 2:**
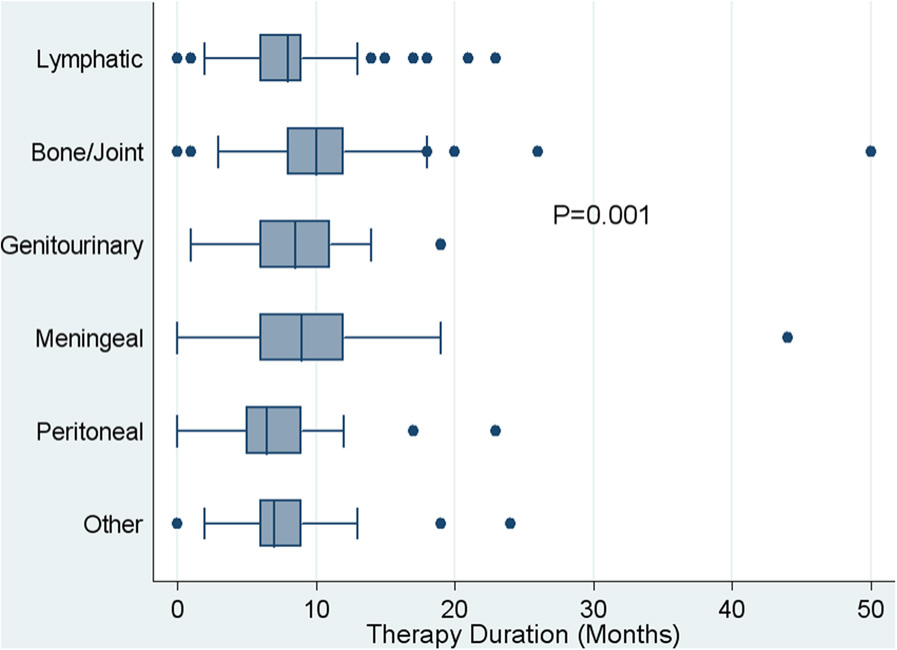
Distribution of therapy duration by type of extrapulmonary TB disease.

There were 9,441 patients diagnosed with tuberculosis in Texas during the entire study period. Of these 837 (16%) had EPTB, and 438 (52%) fulfilled study criteria (Figure 1). In 328 (75%) patients the diagnosis was microbiologically proven. The initial drug regimens used and reported for all study patients were consistent with standard CDC recommendations. The patient characteristics are shown in Table 1. Data on patient weight was missing in many records, and when available not verifiable. Thus, although this factor had been one of the several that prompted the study, it was not further analyzed. Homelessness and substance abuse were common social factors associated with tuberculosis in this cohort (Table 1). The table shows that patients’ median age significantly differed across the type of EPTB (*p = 0.02)*. In addition, EPTB syndrome (i.e., anatomic site of tuberculosis) significantly differed by self-identified “race”: 83% of genitourinary system-tuberculosis patients were “white” while 17% were distributed among the remainder. The median study follow-up period for the entire patient cohort was 7.8 (inter-quartile range: 6.0-10.1) years, and was not significantly different between major disease sites (*p* = 0.11); patients were evenly distributed across the surveillance period. The median therapy duration was 8 (interquartile range: 6-11) months. However, therapy duration differed significantly between EPTB syndromes, especially due to the longer duration of therapy in patients with bone/joint disease (Figure 2).

**Table 1:** Demographic and clinical characteristics at the time of starting therapy for the 438 study patients

**Variable**	** Total**	**Major site of tuberculosis disease**						**p-value**
	**N=438 (%)**	**Lymph nodes n=163(%)**	**Bone and joint n=92 (%)**	**GUS n=24 (%)**	**Meninges n=55 (%)**	**Peritoneum n=43 (%)**	**Other n=61 (%)**	
**Age (years)**	49(18)	47(18)	52(19)	48(17)	43(15)	49(16)	52(19)	*0.02*
**Male**	224(51)	76(47)	46(50)	15(63)	31(56)	23(54)	33(54)	0.63
**Race**								0.02
White	216(49)	73(45)	46(50)	20(83)	24(44)	21(49)	32(53)	
Black	213(49)	85(52)	45(49)	3(13)	31(56)	22(51)	27(44)	
Other	9(2)	5(3)	1(1)	1(4)	0	0	2(3)	
**Ethnicity**								0.39
Hispanic	123(28)	42(26)	25(27)	11(46)	14(25)	11(26)	20(33)	
Non-Hispanic	315(72)	121(74)	67(73)	13(54)	41(75)	32(74)	41(67)	
**Residence**								
Jail	29(7)	14(9)	5(5)	1(4)	2(4)	2(5)	5(8)	0.83
Nursing home	18(4)	8(5)	2(2)	1(4)	3(5)	1(2)	3(5)	0.86
Homeless	13(3)	3(2)	1(1)	1(4)	4(7)	1(2)	3(5)	0.19
**Substance abuse**								
Alcohol	48(11)	17(10)	8(9)	2(8)	3(5)	11(26)	7(11)	0.07
Injection	15(3)	4(2)	6(7)	0	1(2)	4(9)	0	0.06
Other non-injection	35(8)	18(11)	5(5)	2(8)	2(4)	5(12)	3(5)	0.33
**HIV-infected**	93(21)	52(32)	5(5)	1(4)	16(29)	6(14)	13(21)	<0.001
**Prior TB**	15(3)	3(2)	4(4)	3(13)	1(2)	2(5)	2(3)	0.15
**Acid-fast bacilli stain**	74(17)	37 (23)	11 (12)	6(25)	4(7)	6(14)	10(16)	<0.001
***Mtb *** **culture**	308(70)	131(80)	72(78)	16(67)	22(40)	30(70)	37(61)	<0.001
**Completion of therapy**								0.08
Completed	346(79)	133(82)	74(80)	21(88)	36(65)	33(77)	49(80)	
Not completed	53(12)	15(9)	12 (13)	3(13)	8(15)	5(12)	10(16)	

**Figure 3:**
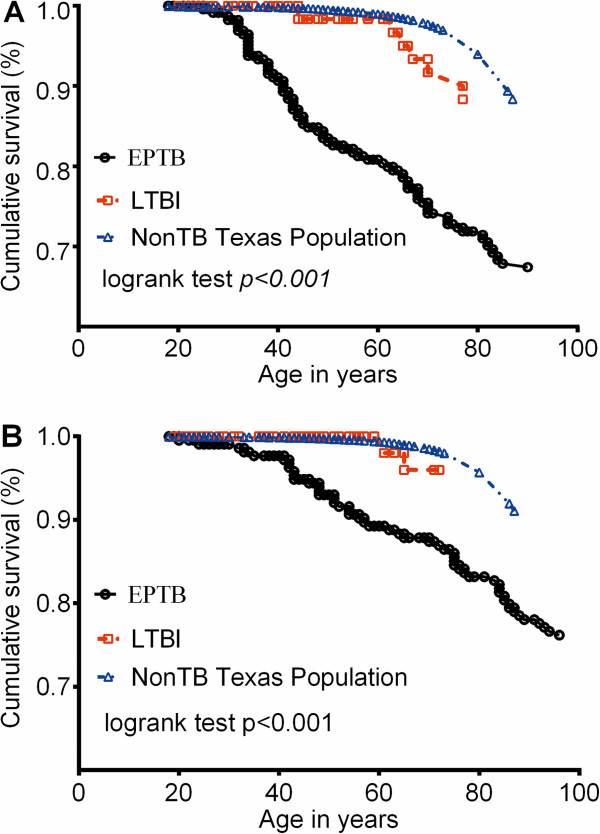
**Cross sectional comparison of survival rates between patients with extrapulmonary TB and controls. (A)**. Men. **(B)** Women. We used two control groups, patients treated for latent tuberculosis, and the general Texas population during the same time periods.

One hundred twenty-four (28%) patients died during the observation period. The overall all-cause mortality rate was 0.025 (95% confidence interval [CI]: 0.021-0.030) per 100 person-year of follow-up; 103 (83%) died within 70.5 months of diagnosis, only 40 (9%) died during therapy, 346 (79%) were recorded as having completed therapy. In the remaining 12% of patients, the reason for failing to document completion of therapy was unknown in 30 patients, 14 were lost to follow up, five moved out of the Texas jurisdiction, and three were uncooperative with healthcare workers. Thus, further analysis was confined to the 346 patients with documented evidence of having completed treatment. Since life expectancy is higher in women than men, and long-term survival is affected by age, the long-term survival in patients with EPTB is shown for different genders and for different ages in Figure 3.

CART analyses identified age-at-diagnosis (age), therapy duration, EPTB syndrome (i.e., anatomic site of disease), and HIV-status as significant predictors of survival. The highest decision node (i.e., root node) ranked by CART was age, with a cut-off point of 64.5 years. Altogether 35/70 (50%) patients >64.5 years died versus 36/376 (13%) patients ≤64.5 years. In patients >64.5 years, the next and last decision node was treatment duration, with a cut-off value of 6 months, with survival of 100% in those with therapy duration ≥6 months versus 43.5% for therapy duration <6 months. Thus therapy duration was the next most important predictor of outcome in patients >64.5 years. On the other hand, for patients ≤ 64.5 years the next decision node was type of EPTB syndrome, with a split into those with genitourinary tuberculosis-TB showing 100% survival versus the rest who had an 83.3% survival. Further down the tree in this younger group of patients, the next most important node was treatment duration among patients without genitourinary tuberculosis, with a cut-off value of 7 months which classified patients into those treated for ≤7 months who had a higher survival of 90.4% versus 81% in those treated for longer than 7 months. In other words, therapy duration versus survival was described by a “V” shaped response. The predictive accuracy for the decision tree, in cross-validation in a test sample, was 92%.

**Figure 4:**
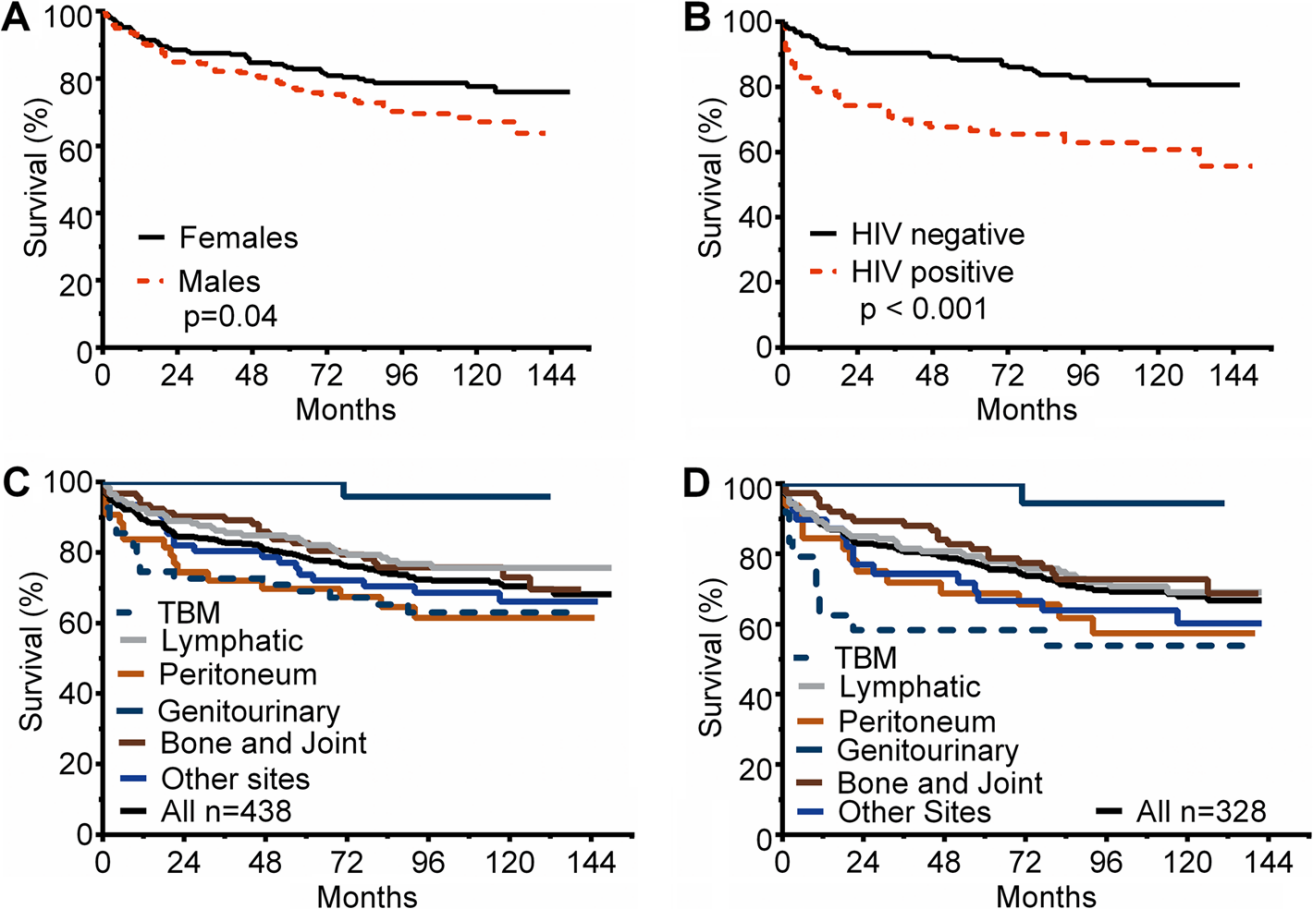
**Survival curves of patients who had risk factors of higher mortality. (A)**. Women versus men. **(B)**. Survival curves for HIV-infected versus non-HIV infected patients. **(C)**. Survival curves of different EPTB syndromes for the entire cohort. **(D)**. Survival curves for different EPTB syndromes for EPTB patients with microbiologically proven disease.

Next, we examined if each of the four parameters identified by CART analyses were also significant when standard parametric multivariate analysis was utilized, with time-to-death as an outcome. The analysis confirmed CART findings, and in addition demonstrated that gender was also a significant risk factor (Table 2). The relationship between gender and mortality is shown in Figure 4A. As regards to age, the hazard ratio for each year of age-at-diagnosis versus time-to-death was 1.05 (CI: 1.04-1.06; p < 0.001). Figure 4B shows the survival curves of HIV-infected versus non-HIV-infected among the 280 patients who were tested for HIV. Figure 4C and D show that when the relationship between EPTB syndrome and time-to-death was examined, genitourinary tuberculosis differed significantly from all other syndromes. When tuberculous meningitis, which had the sharpest early decline in survival after completion of therapy, was examined as the referent, the mortality also differed from some of the non-genitourinary tuberculosis patients (Table 2). Thus, there are 3 significantly different survival categories, namely (a) patients with genitourinary tuberculosis who did as well as those with latent tuberculosis treated with isoniazid prophylaxis, (b) patients with tuberculous meningitis and peritoneal tuberculosis who had very poor long-term outcomes, and (c) an intermediate mortality group comprised of bone/joint, lymphatic, and “other” tuberculosis syndromes.

**Table 2:** Multivariate analysis of factors associated with time to death for the study cohort (402 patients) with available data for the entire follow-up period*

**Variable**	**Hazard ratio (95% CI)**	**P-value**
**Age (years)**		
**18 – 24**	Referent	
**25 – 34**	2.75 (0.60, 12.59)	0.19
**35 – 44**	2.08 (0.46, 9.44)	0.34
**45 – 54**	2.42 (0.54, 10.85)	0.25
**55 – 64**	2.02 (0.42, 9.66)	0.38
**65 – 74**	3.71 (0.77, 17.79)	0.10
**>75**	7.43 (1.54, 35.93)	**0.01**
**HIV-infected versus non-infected**	2.16 (1.22, 3.83)	**<0.01**
**Male**	1.52 (1.01, 2.72)	**0.04**
**TB disease site**		
**Meningeal**	Referent	
**Lymphatic**	0.50 (0.27, 0.91)	**0.02**
**Bone/joint**	0.42 (0.21, 0.81)	**0.01**
**Peritoneal**	0.59 (0.27, 1.31)	0.20
**Other sites**	0.47 (0.23, 0.97)	**0.04**
**Therapy duration**		
**6-months**	Referent	
**<6-months**	2.72 (1.34, 5.50)	**<0.01**
**7 – 10 months**	1.63 (0.85, 3.12)	0.14
**>10-months**	1.52 (0.75, 3.10)	0.25

*Multivariate regression analysis drops patients with missing variables, so the final model comprised of 402 instead of 438 patients.

**Figure 5:**
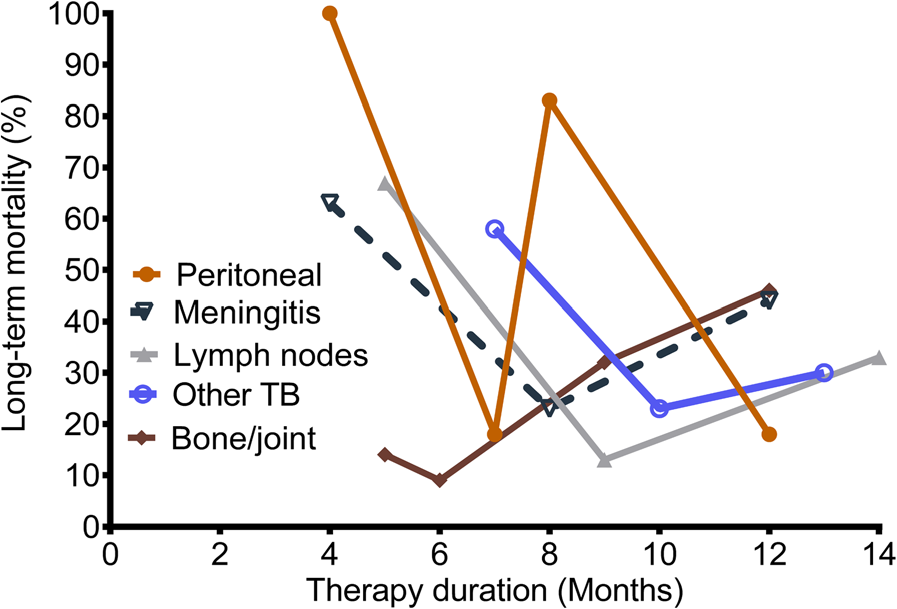
**The relationship between treatment duration and mortality is a “V” shaped curve.** CART analysis was used to identify the treatment duration thresholds associated with long-term outcome within each EPTB syndrome. For all disease syndromes, mortality decreased with longer treatment duration until a nadir, which was the treatment duration associated with lowest proportion of patients with adverse long-term outcomes. Thereafter, mortality increased with longer duration of therapy.

The relationship between treatment duration and mortality was examined in greater detail for each EPTB syndrome, since treatment duration is potentially modifiable. Genitourinary tuberculosis was excluded since only one patient with that diagnosis died. The CART-derived threshold for treatment duration versus long-term mortality is shown in Figure 5. It can be seen in Figure 5, that the relationship between treatment duration for those who completed therapy versus mortality was a “V” shaped curve. As therapy duration increased towards the nadir, survival improved to a maximum at the vertex, after which longer therapy was associated with worse survival. The nadir of the relationship is the therapy duration associated with lowest mortality, which differed between disease syndromes. The therapy duration associated with lowest mortality was 7 months for peritoneal tuberculosis, 8 months for meningitis, 9 months for lymphadenitis, and 10 months for all “other”-tuberculosis syndromes.

## Discussion and conclusion

The pulmonary system has always been considered the “seat” of tuberculosis since the early days of systematic medicine [28-30]. Detailed studies of the metabolic and biochemical status of *M. tuberculosis,* as enumerated in the three population model hypothesis, with direct consequences on therapeutics, have been painstakingly mapped for pulmonary tuberculosis [29,31-36]. Nothing of a similar scope has been performed for most “EPTB”. Our first major finding is that the use of this wastebasket term conceals radically different types of tuberculosis in terms of long-term outcomes and duration of therapy. Lumping these diagnoses together as “EPTB” is no more useful than lumping *Staphylococcus aureus* vertebral osteomyelitis, carbuncles, and brain abscesses as “non-bacteremic *Staphylococcal* disease”. We found a high case fatality rate for some syndromes during the observation period, despite “adequate” chemotherapy. To put this in perspective, the 5 year case fatality rate for *untreated* pulmonary tuberculosis that is smear negative but culture positive is 12-15%, while that for sputum positive is 42-70% [37]. Thus for microbiologically proven and adequately treated meningitis long-term mortality of 46.2% and 42.6% for peritonitis, long-term outcomes are reminiscent of *untreated* “consumption.” We propose that each EPTB syndrome is important in its own right, so that each EPTB syndrome’s pathology, *M. tuberculosis* physiology, and various pharmacological factors should be separately delineated from the others.

It is unclear why TB infection in different organs is associated with different mortality. This could reflect the impact of tuberculosis damage on different organs. An example are the cerebrovascular accidents in tuberculous meningitis [38,39]. Alternatively, this could reflect the differential penetration of antibiotics into different anatomic sites. Poor penetration would reduce both peak concentration and 24 hr area under the concentration-time curve (AUC) to minimum inhibitory concentration ratios, which are known to correlate with microbial kill and suppression of acquired drug resistance in pre-clinical hollow fiber system of tuberculosis studies and in patients [21,40-44]. As an example, in tuberculous meningitis, the blood-brain barrier variably and unpredictably decreases drug levels depending on compartment and drug selected, with such drugs as rifampin achieving only 2-20% those achieved in the serum, while isoniazid AUC is 80% that in serum [10,45,46]. Indeed, when drugs that penetrate well into the meninges such as quinolones are included, or rifampin’s dose is increased so that the AUC is increased, tuberculous meningitis patients have decreased mortality [8,10]. Similarly, in bone/joint disease, the percentage of drug in foci in sclerotic bone is 0-8% for rifampin, 0-6% for pyrazinamide, and 0-23% for isoniazid [47]. These drug-concentration scenarios are known to lead to acquired drug resistance and poor efficacy [21,48]. Finally, it could be that for some syndromes such as meningitis, the *M. tuberculosis* genotypes that preferentially cause such disease are more difficult to kill and have a higher propensity to fail and develop drug resistance [49-52].

A third important finding was that the relationship between therapy duration for each syndrome versus outcome as “V” shaped. This implies that there is a combination therapy duration point associated with the best long-term outcome, and that therapy duration beyond this point leads to poorer survival. Thus, unnecessarily long therapy could be harmful to patients. In studies of antibiotics used to treat other bacterial infections, it has been suggested that better outcomes occur with shorter rather than longer duration therapy [53]. However, this is a matter of ongoing debate. In our hollow fiber studies, longer duration of isoniazid and pyrazinamide therapy was an important driver of acquired drug resistance by *M. tuberculosis*[41,42]. Thus, longer therapy duration than necessary could worsen microbiologic outcomes. Nevertheless, given the retrospective nature of the data, we cannot definitively ascribe causation of poor long-term outcomes to the longer therapy duration. Another possibility is that patients who were not doing well could have had their therapy duration extended by clinicians, so that the longer therapy duration may be an indication of patients who were having poor outcome rather than being the cause of poor outcome, a *post hoc ergo propter hoc* fallacy. These possibilities cannot be teased out in a retrospective study, and thus causation cannot be ascribed at the present moment. Our findings should thus be viewed merely as generating a hypothesis, which must then be tested in prospective studies.

Our study has several limitations. First, the most important limitation is the retrospective nature of the design. Additionally, since the data were routinely collected for surveillance purposes some data points were missing, while some could have been entered erroneously. Second, the study patients’ age distribution was skewed in favor of an elderly population with naturally higher co-morbid conditions and consequently poorer long-term survival. Third, variations in the combinations of anti-tuberculosis drugs used to treat each patient could affect outcomes, given comorbid conditions with potential drug interaction, or regimen changes to accommodate acquired drug resistance, all of which limit interpretation of the data. Fourth, a more detailed exploration of the factors identified as associated with overall mortality in treated tuberculosis patients was precluded by the incompleteness of data in the Report of Verified Case of Tuberculosis. Finally, in the current analysis, we chose to use CART analyses. However, CART is prone to over fitting, and slight changes in dataset may lead to selection of different predictors. This problem led to extension of the procedure into random forests, where instead of the forest instead of a single tree, is grown [54]. We utilized CART because it is more intuitive to clinical decision making by clinicians, is familiar to clinicians, and is much easier to interpret. In order to make sure the predictors chosen by CART were indeed correct and not due to instability, standard parametric multivariate analysis was utilized to examine the CART-derived predictors and thresholds, which were all found to be statistically significant.

In summary, different EPTB syndromes have different long-term outcomes and different therapy durations associated with lowest mortality. The long-term outcomes for such syndromes as peritoneal and meningeal tuberculosis adequately treated with modern chemotherapy are sufficiently poor as to be indistinguishable from some types of tuberculosis before the advent of chemotherapy. The different syndromes should not be lumped into single wastebasket diagnosis of EPTB.

## Competing interests

TP, JGP, RGH- no conflict of interest. TG has received research grants from Merck for work on antifungal agents; TG also founded and owns Jacaranda Biomed, Inc.

## Authors’ contributions

TP conceived the study, performed the database search, performed data analysis, and drafted the manuscript. JGP helped with search in database, performed statistical analysis, performed classification and regression tree analysis, and drafted the manuscript. RGH: conceived the original study, was the principal investigator for IRBs, participated in its design and coordination, and drafted the manuscript. TG conceived of the study, and participated in its design and coordination, supervised the entire study process, performed statistical analysis, helped to draft the manuscript, and performed final editing. All authors read and approved the final manuscript.

## Pre-publication history

The pre-publication history for this paper can be accessed here:

http://www.biomedcentral.com/1471-2334/14/115/prepub
